# Influence of (Sub) Structure Development within Rotary Swaged Al–Cu Clad Conductors on Skin Effect during Transfer of Alternating Current

**DOI:** 10.3390/ma15020650

**Published:** 2022-01-15

**Authors:** Lenka Kunčická, Radim Kocich, Petr Kačor, Michal Jambor, Miroslav Jopek

**Affiliations:** 1 Institute of Physics of Materials, Czech Academy of Sciences, Žižkova 22, 61600 Brno, Czech Republic; jambor@ipm.cz; 2 Faculty of Materials Science and Technology, Vysoká Škola Báňská–Technical University of Ostrava, 17. listopadu 2172/15, 70800 Ostrava, Czech Republic; 3 Department of Electrical Power Engineering, Vysoká Škola Báňská–Technical University of Ostrava, 17. listopadu 2172/15, 70800 Ostrava, Czech Republic; petr.kacor@vsb.cz; 4 Faculty of Mechanical Engineering, Brno University of Technology, Technická 2, 61669 Brno, Czech Republic; m.jopek@fme.vutbr.cz

**Keywords:** alternating current, *skin effect*, rotary swaging, clad composite, microstructure

## Abstract

The nature of alternating current transfer via metallic materials is specific, since the current density tends to be inhomogeneous across the cross-section of the conductor and the *skin effect* tends to occur. However, the influence of this effect on the behaviour of the conductor can be optimized via the design and fabrication procedures. The study presents innovative design of an Al–Cu clad conductor, which is supposed to affect favourably the influence of the *skin effect*. The clad conductors of various diameters (20 mm, 15 mm, and 10 mm) were fabricated via rotary swaging at room temperature, and their electric characteristics were subsequently examined both experimentally and via numerical simulations. Structure analyses performed to document the effects of the swaging technology on the development of substructure and characteristic structural features were carried out by scanning electron microscopy (electron backscatter diffraction analyses), and transmission electron microscopy. The results showed that the design of the composite has a favourable effect on decreasing the power losses during alternating current transfer and that the substructure development affected favourably the electric resistance of the conductor. The highest electric resistance was measured for the composite conductor with the diameter of 20 mm (1.8% increase compared to electric resistance during transfer of direct current). This value then decreased to 0.6%, and 0.1% after swaging down to the diameters of 15 mm, and 10 mm; the 10 mm composite featured the finest grains, partially restored structure, and texture randomization compared to the 20 mm and 15 mm composites. Manufacturing of the clad composite via rotary swaging imparted advantageous combinations of both the electric and mechanical properties, as swaging also introduced increased microhardness.

## 1. Introduction

Clad composites consisting of metallic components, sometimes also denoted as metallic laminates, are gaining increasing attention throughout numerous industrial branches (automotive, marine, aerospace, etc.) and typically consist of two or more elements, for example Al plus Mg [1], Ni [2], or Sn [3], Cu plus Ti [4], Ni [5], or steel [6], combinations of Al and Cu plus Mg [7], or Zn [8], and more. However, probably the most popular is the combination of Al plus Cu. Both the metals exhibit excellent thermal and electric conductivity, and advantageous resistance to corrosion. Moreover, compared to commercially pure (CP) Cu, composites consisting of Al and Cu combinations generally have lower cost and lighter weight [9]. Clad composites can quite easily be manufactured by welding methods (e.g., explosive welding [10], diffusion welding [11], or friction stir processing [12]). Nevertheless, welding is characterized by (localized) effects on structural characteristics, which can be deteriorating for Al–Cu clad composites as they tend to form brittle intermetallic phases at mutual interfaces at processing temperatures higher than 350 °C [13,14]. Moreover, both the Al and Cu have a high tendency to dissolve oxygen at elevated temperatures, which can significantly deteriorate the electric conductivity of a final composite conductor. Therefore, fabrication by welding is not suitable for these materials.

Manufacturing metallic clad composites by methods based on imposing high shear strength, i.e., methods of severe (and intensive) plastic deformation, is promising since they can advantageously be performed also at room temperature, and are highly effective from the viewpoint of bonding of the individual metallic layers. Generally, the quality of mutual bonding is affected by the amount of the imposed shear strain. Therefore, severe plastic deformation (SPD) methods, such as high pressure torsion [15], and methods based on equal channel angular pressing (ECAP) [16,17] (ECAP with partial back pressure [18], ECAP-Conform [19], twist channel angular pressing [20], twist channel multi angular pressing [21], etc.) are considered to be very favourable. However, most of the SPD methods are discontinuous and are designed to process small bulk samples. For this reason, researchers also advantageously use other processing methods based on imposing high shear strain into the processed materials leading to production of well-bonded composites featuring advantageous fine-grained structures (e.g., combinations of stir casting and cryorolling [22], or repeated press and rolling [23]). Another method of intensive plastic deformation favourable for production of various modern materials from powder-based compounds, through composites, to metallic laminates, is rotary swaging [24,25,26]. Not only can swaging be used to fabricate long axisymmetric composite products (e.g., conductors), but the compressive stress state dominating during processing is also advantageous for mutual bonding of composite layers.

The fabrication technology, together with applied processing conditions (i.e., temperature, stress state, imposed shear strain, activated strain paths etc.), clad composite design, and selected composite components affect non-negligibly the deformation behaviour and structure characteristics of the composite, which goes hand in hand with the final mechanical, utility, and electrical properties of the product. Generally, the electric resistivity of a conductor is affected by its material, dimensions, and operational temperature. Considering constant operational temperature, the resistivity of the conductor is primarily dependent on the used materials and their dimensions, i.e., composite design. For electro-conductive composites, determining the intended application of the product before its design and production is also favourable. In other words, transfer of direct and alternating current feature characteristic differences, which can advantageously be considered during the composite design procedure. Transfer of alternating current (AC) through a conductor induces the development of electromagnetic waves, which substantially affect the distribution of current density across the conductor cross-section. Consequently, the current density is primarily concentrated beneath the conductor‘s periphery (to a certain depth in which the current density decreases). This phenomenon is characterized as the *skin effect*.

Previously published works dealing with Al plus Cu composites mostly focused on their design [27,28], fabrication methods [14,29], deformation behaviour [30], structure characterization [31,32,33], and determination of mechanical properties [34,35]. Several studies also dealt with basic characterization of their electric properties [13]. Nevertheless, despite the fact that some researchers investigated AC characteristics for other types of composites (e.g., composites with carbon nanofibers [36], or polymer-based ones [37]) studies focused on the characterization of AC behaviour while being transferred through Al–Cu clad conductors, are not available (to the best of the authors’ knowledge). For this reason, the presented study focuses on characterization of the electric conductivity of innovative self-designed Al–Cu clad conductors fabricated via room temperature rotary swaging during AC transfer. In the study, we especially focused on the effect of deformation ratio and the induced structure characteristics, the analyses of which were carried out by scanning and transmission electron microscopies. Experimental measurements of electric properties were supplemented with numerical simulations of distribution of current density across the cross-sections of the swaged composites.

## 2. Materials and Methods

### 2.1. Preparation of Material

**Figure 1:**
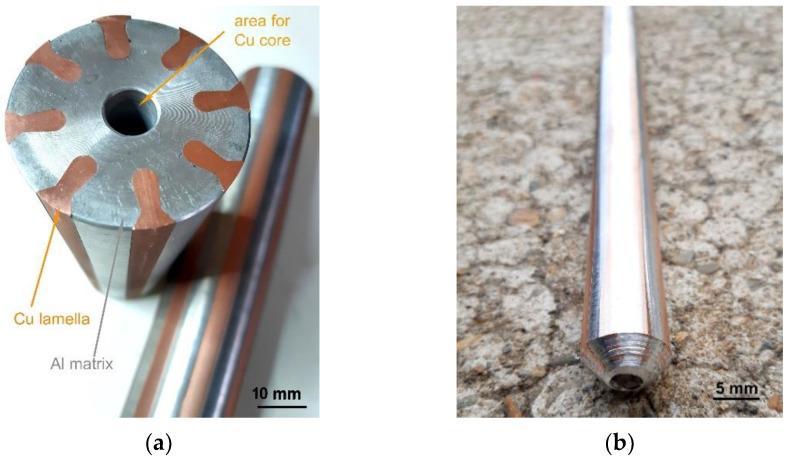
Assembled Al–Cu semi-product before inserting Cu core (**a**); swaged composite rod 10 mm in diameter (**b**).

For the experiments, clad composite rods of a unique self-designed stacking sequence were prepared. Based on our previous research, which revealed that it is advantageous for the composite to contain Cu primarily in its axis and periphery [14,27], they consisted of Al matrix (electro-conductive commercial purity Al with 0.26% Fe, 0.22% Si and 0.04% Cu) of 50 mm in diameter, eight peripheral Cu lamellas and a Cu core with 10 mm in diameter (electro-conductive commercially pure Cu with 0.014% P, 0.002% O, and 0.002% Zn) inserted into this (see Figure 1a for the assembled semi-products before inserting the Cu core).

The assembled Al–Cu semi-products were further rotary swaged at room temperature from their original diameters of 50 mm to a rod with the final diameter of 15 mm, and to a rod with the final diameter of 10 mm, the photo of which is depicted in Figure 1b. The imposed swaging degrees, calculated using Equation (1), were 2.4 and 2.8 for the rods swaged to the diameters of 15 mm, and 10 mm, respectively.

(1) $$\varphi =\ln\frac{S_{0}}{S_{n}}$$

where $S_{0}$, $S_{n}$ are cross-sectional areas of the Al–Cu composites at inlet and outlet from swaging dies, respectively.

### 2.2. Experimental Determination of Electric Characteristics

The basic material parameter, which needs to be determined to characterize the electric behaviour of a material, is the specific electric resistivity ρ (Ω·m), or specific electric conductivity $\sigma =\frac{1}{\rho }$ (S·m^−1^). The value of the specific resistivity depends on the geometry of the conductor, i.e., on its cross-section *A* (m^2^), and length *L* (m), both of which need to be constant in order to calculate the resistivity *R* (Ω), for which Equation (2) can be used.

(2) $$R=\rho \cdot \frac{L}{A}$$

Equation (2) takes into account homogeneous distribution of current density *J* (A·m^−2^) across the conductor (composite) cross-section *A*. For direct current (DC) transfer, i.e., transfer of current *I* (A) through a conductor of constant cross-section *A* and length *L*, the basic condition of homogeneous distribution of current density *J* is met. Therefore, the simple Equation (3) can be used for DC transfer throughout a conductor (composite):

(3) $$J=\frac{I}{A}$$

Nevertheless, as mentioned in Section 1, by the effect of the induced magnetic wave, AC transfer features inhomogeneous distribution of current density *J* across the cross-section of the conductor (composite). The level of its inhomogeneity can be characterized by the parameter of skin depth δ (m), which is defined as a radial distance from conductor surface towards its axis. At this distance, damping of the electromagnetic wave is equal to *1/e*, where *e* is the Euler number, which is approximately 37%. The electromagnetic wave causes the current density *J*, which is otherwise distributed homogeneously across the conductor cross-section, to concentrate in its outer edge, which results in the occurrence of the aforementioned *skin effect*. Generally, transfer of AC affects the overall resistivity *R* of a conductor in all cases, however, the influence of the *skin effect* is evident especially for conductors the diameter *D* of which is larger than 2·δ. The skin depth δ can be calculated according to Equation (4),

(4) $$\delta =\sqrt{\frac{2\cdot \rho }{\omega \cdot \mu }}=\sqrt{\frac{\rho }{\pi \cdot f\cdot \mu_{0}\cdot \mu_{r}}}$$

where ω (rad·s^−1^) is angular frequency, μ (H·m^−1^) is magnetic permeability of measured material, f (Hz) is frequency of supplied current, $\mu_{0}$ (H·m^−1^) is permeability of vacuum, and $\mu_{r}$ (-) is relative permeability.

Equation (4) demonstrates that the skin depth δ is directly proportional to the specific electric resistivity *ρ* of material, and inversely proportional to the current frequency f and permeability *μ* of material. In other words, the skin depth decreases with increasing frequency and/or permeability, and increases with increasing specific electric resistivity.

**Figure 2:**
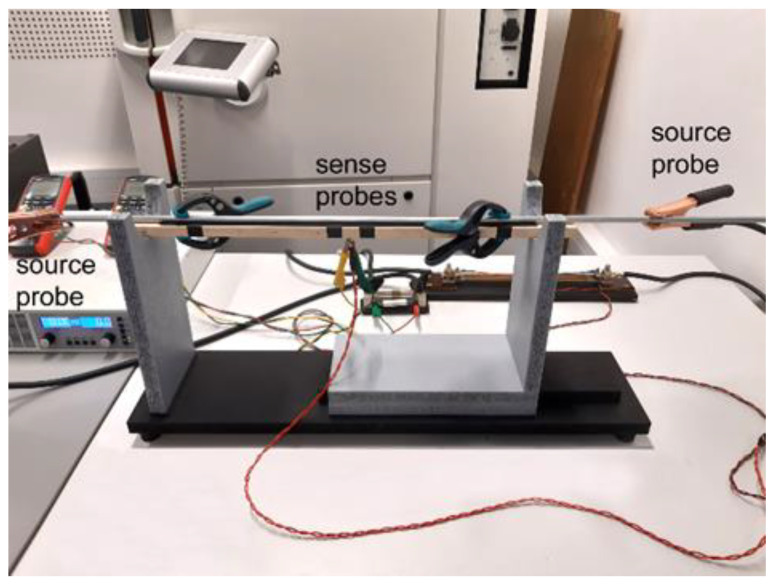
Experimental setup for electric measurements with depicted locations of sense and source probes.

Having prepared the composite rods, electric characteristics were experimentally examined. The four-wire resistance measurements method was used to perform highly accurate measurements of the electric resistivity for the 15 mm and 10 mm swaged composite rods, as well as rods of the original Al and Cu. In this method, the sense probes (a pair of electrodes) are used to measure the voltage drop on selected length of the swaged composite, while the source probes (another pair of electrodes) are used to supply current for the measurements [38] (see Figure 2 for the experimental setup). By using two individual pairs of electrodes the supplied electric current is separated from the measurements. The measured composite lengths were *L* = 500 mm for both the 15 mm and 10 mm diameter rods. Therefore, for monitoring the voltage drop during AC transfer, the sense probes were mounted on the swaged composites in the distance of *dL* = 500 mm. During the measurements, the monitoring conductors mounted to the voltage sensors have to be kept in the closest possible vicinity of the conductor (composite) surface, since the larger is the distance between the monitoring conductors of the voltage sensors and the surface of the measured conductor, the higher is the probability of the occurrence of additional voltages induced by the electromagnetic wave, which eventually causes measurement errors.

Firstly, the specific electric resistivity of the original materials, i.e., electro-conductive (ETC) Al and Cu, during AC transfer was determined (to be used for further computations). These values are demonstrated in Table 1, which is also supplemented with the values of permeability *μ* for both the original metals. The table shows that Cu is a diamagnetic material, while Al is a paramagnetic one. Last but not least, the table shows the skin depth for both the metals calculated using Equation (4) at the frequency of f = 50 Hz.

### 2.3. Numerical Predictions of Electric Characteristics

**Figure 3:**
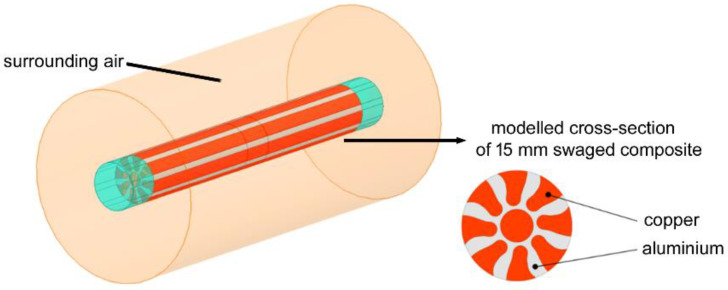
General model of clad composite rods used in numerical simulations, cross-section of 15 mm modelled composite rod shown in detail.

Numerical simulations of AC transfer through the composites were performed for the swaged rods having the diameters of 20 mm, 15 mm, and 10 mm. The basic geometries of the swaged composite rods were based on the experimentally acquired ones, as regards their length, diameter, and distribution of the Al and Cu components across the composites’ cross-sections. Figure 3 shows the general depiction of the used clad composite geometrical model. In the detail of the figure, the cross-section of the modelled 15 mm swaged composite is shown. Among the clad composite rod itself, the model also contained auxiliary components, which served to provide the input and output of AC (see the ending parts of the modelled composite rod depicted in blue). For the purposes of the simulations, as well as to ensure uniform input and output of the supply current, these input and output composite volumes were characterized as conductors featuring negligible conductivity.

The analysis of the electromagnetic model of the Al–Cu clad composite was solved as a harmonic task, which enabled to calculate the value of power losses dP*_AC_* (for known value of transferred current). Once the values of the transfer current I, i.e., the input parameters, as well as the power losses dP*_AC_*, i.e., the output parameters, are known, the overall value of electric resistivity R of the swaged clad composite rods can be determined using Equations (5) and (6),

(5) $$dP=\frac{1}{\sigma }\int_{vol}J^{2}\cdot dV$$

(6) $$R=\frac{dP}{I_{RMS}^{2}}$$

where dV is a volume element of swaged composite (m^3^), and $I_{RMS}^{}$ is root mean square value of supplying AC current.

### 2.4. Analyses of Experimental Material

The analyses of structures of the 15 mm and 10 mm swaged clad composites were performed using scanning and transmission electron microscopies (SEM and TEM). For SEM observations, the electron backscatter diffraction (EBSD) method was primarily used. As for the samples, cross-sectional cuts from the swaged composites were prepared by manual grinding and electrolytical polishing. Subsequently, the samples were observed using a Tescan Lyra 3 XMU FEG/SEMxFIB microscope equipped with Symmetry EBSD detector (Tescan Orsay Holding a.s., Brno, Czech Republic) under the tilt of 70° with the scanning step of 0.5 µm. The analyses were subsequently evaluated using the Aztec Crystal software (Oxford Instruments, Abingdon, UK). The characteristic parameters for the analyses were the max. deviation of 15° for texture observations, and the 15° limit between the low angle and high angle boundary defining grains and sub-grains (grain size and CSL twin boundaries analyses). Detailed substructure analyses were further performed for the Cu lamellas of both the 15 mm and 10 mm swaged composites using a JEM-2100 TEM microscope (JEOL, Tokyo, Japan) operating at 200 kV. The samples for TEM were prepared using a focused ion beam (FIB) assembled on the mentioned Tescan Lyra 3 XMU microscope. The FIB sample preparation consists of gradual milling of a thin lamella with Ga ions to the final thickness of approximately 120 nm.

Last but not least, supplementary analyses of Vickers microhardness were performed for both the 15 mm and 10 mm swaged composites using a Zwick/Roell measuring machine (Zwick Roell CZ s.r.o., Brno, Czech Republic). For each measured point, the indent load was 200 gf, and loading time was 10 s.

## 3. Results

### 3.1. Electric Characteristics

Firstly, the results of harmonic analyses, i.e., electromagnetic numerical simulations, were evaluated. The most important output parameter from the analyses was the distribution of current density *J* across the cross-section of the examined conductors. Before conducting the analyses for the swaged clad composites, basic analyses of the distributions of current density for the original Al and Cu metals were performed (to enable comparison with the swaged rods). These analyses were carried out not only for AC transfer, but also for DC transfer (for comparison).

**Figure 4:**
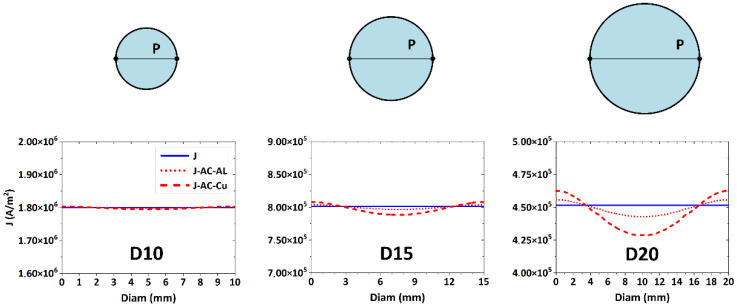
Numerically predicted distributions of current density across cross-sections of 20 mm, 15 mm, and 10 mm Al and Cu conductors.

Figure 4 depicts the predicted distributions of current density for conductors of Al and Cu, with the diameters of 10 mm, 15 mm, and 20 mm, respectively. The current density is depicted along the center line *P*, plotted between two points located on the periphery of the conductor, i.e., representing its diameter. The figure shows that the current density was only minimally deformed for both the conductors with the diameters of 10 mm and thus the *skin effect* can be neglected for this conductor diameter. In other words, the current density lines for AC transfer through Al and Cu conductors were aligned with the line representing the current density during DC transfer. For the conductors with the diameters of 15 mm, the *skin effect* was manifested by a variable current density. Its values were higher at the peripheries of both the Al and Cu conductors and then decreased towards their axes. The *skin effect* was more pronounced for the Cu conductor. Finally, the 20 mm diameter Al and Cu conductors, the *skin effect* for which was obvious, were examined. As seen in Figure 4, the current density line was heavily deformed for the Cu conductor. This phenomenon occurred due to its higher electrical conductivity and smaller penetration depth of electromagnetic wave (compared to Al) [39]. At the periphery of the conductor, the current density was about 3% higher than the current density for DC transfer, and gradually decreased towards its axis to 94% of the DC current density value.

**Figure 5:**
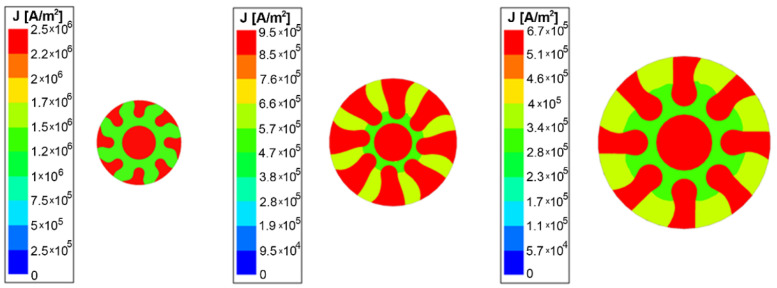
Numerically predicted distributions of current density across cross-sections of 10 mm, 15 mm, and 20 mm swaged composite conductors (cross-sectional composites’ geometries based on experimental observations).

The predicted distributions of current density for the swaged clad composites of the diameters of 10 mm, 15 mm, and 20 mm, respectively, are depicted in Figure 5. As can be seen from the figure, the current density is generally higher in the Cu components of the clad composites (compared to the Al matrix). As regards the homogeneity of the cross-sectional current density distribution, the occurrence of deformations of current density lines, i.e., the *skin effect*, was observed primarily in the Al matrix of the composites, especially for greater diameters of the clad conductors. In other words, the *skin effect* started to occur from the composite diameter of 15 mm. Whereas the Al matrix is distributed homogeneously across the entire cross-section of the swaged composites, its Cu components are distributed in individual segments, i.e., lamellas and core. Nevertheless, the behaviour is different compared to (commercially) pure metals. For conductors consisting of pure metals, the current density is distributed with respect to the conductor diameter. On the other hand, for a composite conductor consisting of a combination of metals, i.e., Al and Cu in this case, not only the fact that the values of current density are higher in the Cu components, but also the influence of the skin depth in its Al component, is evident.

**Figure 6:**
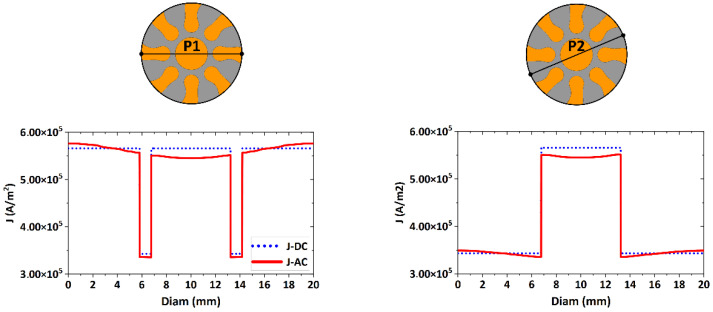
Distributions of current density for 20 mm swaged composite conductor along paths *P1* and *P2* across the conductor diameter.

The skin depth for Cu corresponds to *δ_Cu_* = 9.41 mm, and for Al it corresponds to *δ_Al_* = 12.07 mm. Therefore, the effect of the deformations of current density lines has to be concentrated primarily in the Al component of the clad conductor. This phenomenon is evident for the swaged composites with diameters larger than 15 mm, i.e., the 15 mm and 20 mm swaged rods. The deformation of current density is thus the most evident for the clad composite with the diameter of 20 mm, a region with a higher current density value which is formed around the outer edge of the conductor. The current density distribution for the 20 mm swaged composite, the *skin effect* for which was the most pronounced, was also plotted across the *P* line characterizing the diameter of the conductor (similar to the analyses performed for the individual Al and Cu conductors depicted in Figure 4). For this analysis, two measurement paths were chosen, labelled as *P1* and *P2* lines (Figure 6). The *P1* line passes through the Cu lamellas, Al matrix, and Cu core, while the *P2* line passes through the Al matrix and Cu core, excluding the Cu lamellas. As can be seen in Figure 6, the current density distribution along the *P1* and *P2* paths for the 20 mm diameter clad composite was significantly deformed due to the *skin effect* and the deformation of the current lines started to appear within the Al matrix of the clad composite conductor.

Experimental determination of the electric characteristics of the clad conductors for AC transfer was carried out according to the methodology described in Section 2.2. Firstly, measurements and calculations of specific resistivity *ρ* and resistance *R* for the individual Al and Cu conductors were performed. The values of specific resistivity and resistance, which were subsequently considered when evaluating electric characteristics of the swaged clad composites, were 28.772 × 10^−9^ Ωm and 441.3 × 10^−6^ Ω, respectively, for Al, and 17.468 × 10^−9^ Ωm and 22.51 × 10^−6^ Ω, respectively, for Cu.

The overall effect of increasing deformation of current density on increasing values of power losses *dP_AC_* is depicted in Table 2. The table clearly shows that the values of power losses during AC transfer *dP_AC_* are higher than the values of power losses during DC transfer *dP_DC_*. The table is supplemented with the calculated values of *k_s_* coefficient. Table 2 also documents that the increase in *skin effect* for the clad composites is slower than for ETC copper, but more rapid than for ETC aluminium. This is given by the stacking sequence and characteristic dimensions of the Al and Cu components within the clad composite. For small conductor diameters (10 mm), this effect is more or less negligible. However, as the conductor diameter increases (up to 20 mm), its AC resistance increases by approximately 1% for ETC aluminium, 2.5% for ETC copper, and 1.8% for clad composite.

### 3.2. (Sub) Structure Development

The (sub)structure analyses primarily focused on characterization of sizes and orientations of grains within the individual composite components. The grain size parameter was evaluated via the maximum feret diameter value. This value can be defined as the largest distance between two points characterizing an individual grain [40]. The average grain size values for the individual components of the composites were calculated from the total numbers of grains occurring at the individual scanned regions. The presented grain size distribution charts for the composites’ components then show area weighted fractions of the grains having the respective maximum feret diameter values (*X* axis). In other words, the graphical depictions of grain sizes depict the total areas occupied by the grains having the respective max. feret diameter mentioned at the *X* axis [41].

The analyses showed that incremental swaging introduced gradual grain refinement, as the measured average grain sizes decreased with decreasing composite diameter. Also, the swaged rods exhibited differences in the average grain sizes between the composite peripheral and axial regions, i.e., the peripheral Cu lamellas and axial Cu core. The average grain size for the 20 mm composite Cu lamellas was 8.0 µm, while for the core it was 10.2 µm. The Cu lamellas of the 15 mm composite featured the average grain size of 6.9 µm, whereas for the core it was 9.7 µm. Finally, the average grain size for the 10 mm composite was 5.7 µm for the Cu lamellas, and 8.4 µm for the core.

**Figure 7:**
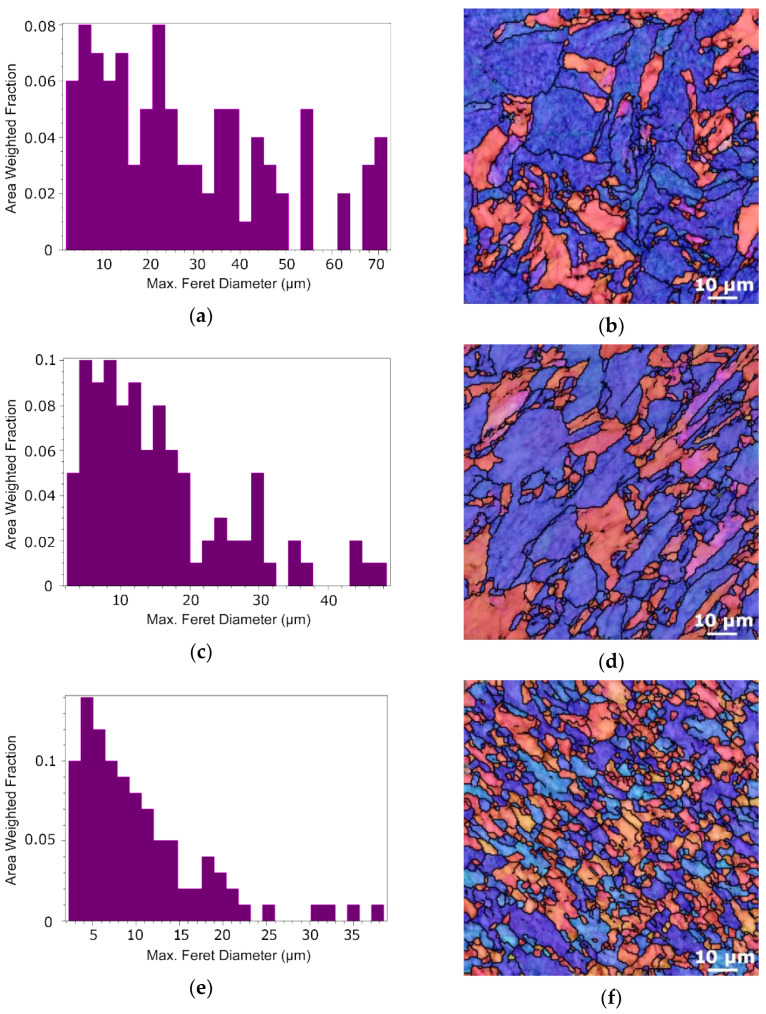

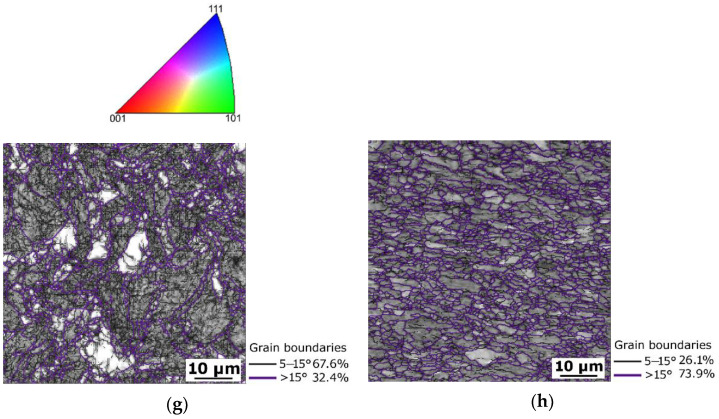
Results of structure analyses for: Cu lamella of 20 mm composite–grain size distribution chart (**a**), orientation image map (OIM) (**b**); Cu lamella of 15 mm composite–grain size distribution chart (**c**), OIM (**d**); Cu lamella of 10 mm composite–grain size distribution chart (**e**), OIM (**f**). Grain boundary analyses for Cu lamella of: 15 mm composite (**g**); 10 mm composite (**h**).

The (sub)peripheral regions of the Cu components evidently featured lower average grain sizes than the Cu cores for all the swaged composite diameters, which can primarily be attributed to the character of the swaging process (the maximum imposed strain can be detected at the periphery of the swaged rod [30]). For this reason, the following figures depict the structure characteristics acquired for the Cu lamellas. The results of structure analyses are graphically documented by the grain size distribution charts and respective structure images depicting orientation image maps (OIMs) for the Cu lamellas of the 20 mm composite (Figure 7a,b), 15 mm composite (Figure 7c,d), and 10 mm composite (Figure 7e,f). The analyses of grains orientations documented that the <001> and <111> grains orientations were prevailing within the lamellas of the 20 mm and 15 mm swaged composites. However, the grains within the lamellas of the 10 mm composite exhibited deviations from these two preferential directions, as the colours of the individual grains varied from the characteristic red and blue. This phenomenon, together with the presence of fine equiaxed grains (Figure 7f) point to the occurrence of restoration processes, i.e., dynamic recrystallization, during swaging to the final diameter of 10 mm. This hypothesis was further confirmed by the analyses of low- and high-angle grain boundaries (LAGBs and HAGBs) performed for Cu lamellas of the 15 mm and 10 mm composites, the results of which are depicted in Figure 7g,h, respectively. The figures show that the fraction of HAGBs increased from 32.4% to more than 70% during the final swaging pass.

**Figure 8:**
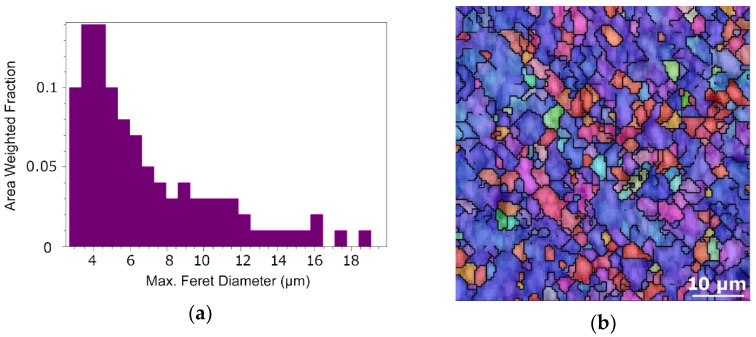

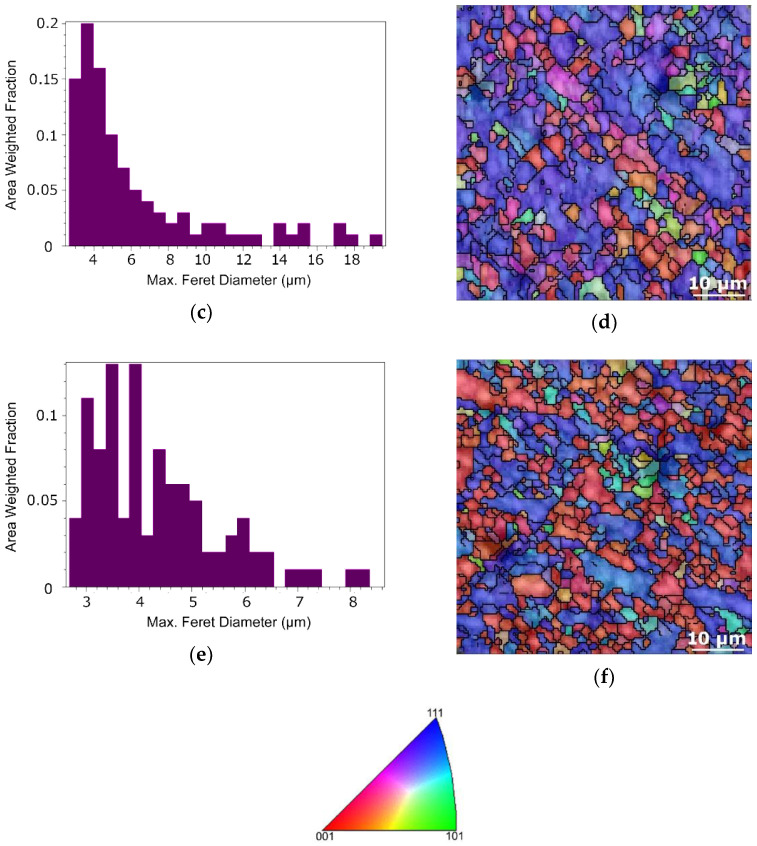
Results of structure analyses for: Al matrix of 20 mm composite–grain size distribution chart (**a**), orientation image map (OIM) (**b**); Al matrix of 15 mm composite–grain size distribution chart (**c**), OIM (**d**); Al matrix of 10 mm composite–grain size distribution chart (**e**), OIM (**f**).

Similar to the Cu composite’s components, structure analyses providing data on the grain sizes and their orientations were performed for the Al matrices (analysed in regions between a Cu lamella and the Cu core for each composite diameter). The chart depicting the area weighted fraction grain size distribution for the Al matrix of the 20 mm composite is shown in Figure 8a, whereas the respective structure with IOM depiction is shown in Figure 8b. The grain size distribution for the 15 mm composite and the respective structure is then depicted in Figure 8c,d. Finally, the grain size distribution and the structure for the Al matrix of the 10 mm swaged composite are shown in Figure 8e,f. The results of the analyses showed that the average grain sizes for the Al matrix of the 20 mm, 15 mm, and 10 mm composites were 4.7 µm, 4.4 µm, and 4.0 µm, respectively. Not only did the 10 mm composite feature the smallest average grain size, but also the smallest standard deviation for this value, as the maximum Al grain size for this composite was 8.4 µm.

**Figure 9:**
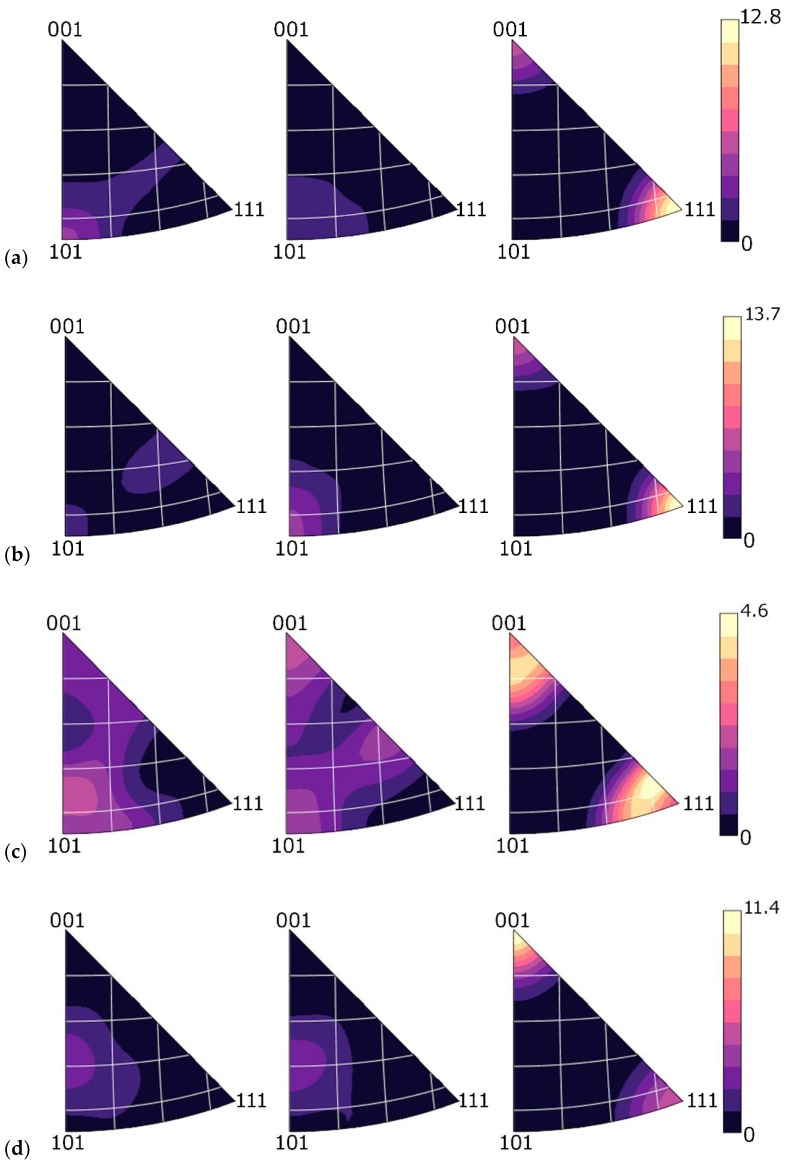

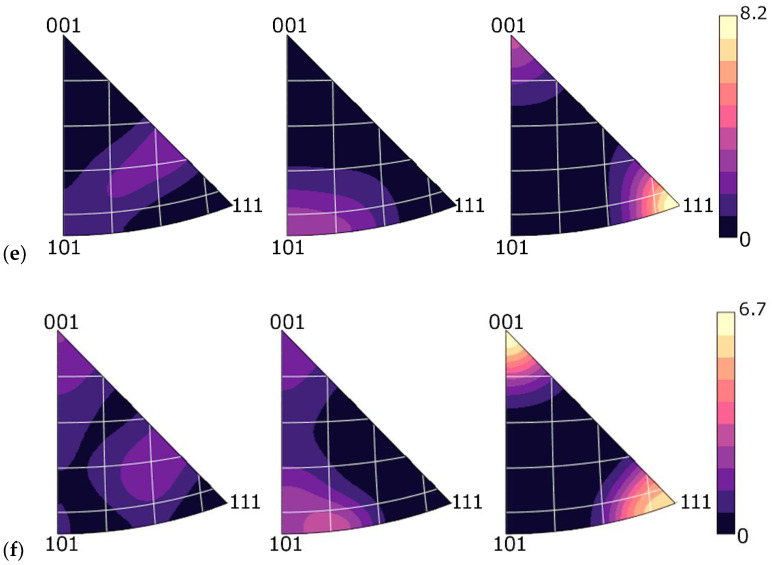
Inverse pole figures (IPFs) for Cu lamellas of swaged clad composites: (**a**) 20 mm; (**b**) 15 mm; (**c**) 10 mm; (**d**) IPFs for Al matrices of swaged clad composites: 20 mm; (**e**) 15 mm; (**f**) 10 mm.

The above characterized results of structure analyses were also supported by the analyses of texture, i.e., detailed preferential grains orientations analyses, the results of which are summarized via the inverse pole figures (IPFs). The IPFs for the Cu lamellas of the 20 mm, 15 mm, and 10 mm composites are depicted in Figure 9a,c, while the IPFs for the Al matrices of the 20 mm, 15 mm, and 10 mm composites are depicted in Figure 9d–f. Evidently, the preferential orientation <111>||swaging direction (SD) prevailed within the Cu lamellas of both the 20 mm and 15 mm composites (Figure 9a,b). Texture intensity was comparably strong for both the conductors. On the other hand, the lamellas of the 10 mm swaged conductor exhibited evident weakening of the maximum texture intensity, and equalization of the intensities of the <111>||SD and <100>||SD preferential fibres (Figure 9c). The grains also featured greater misorientation angles, which is demonstrated by the widening of the circles in the edges of the IPFs. This phenomenon again points to the occurrence of restoration processes during swaging to 10 mm. Figure 9d–f then document that the maximum texture intensity for the Al matrix gradually decreased during the swaging process, and that the intensities of the preferential <111>||SD and <100>||SD texture orientations varied for different composite diameters. The maximum texture intensity decrease was more evident during the first examined swaging step to 15 mm. On the other hand, the final swaging pass contributed to equalization of the maximum intensities of both the <111>||SD and <100>||SD preferential texture fibres.

To confirm the supposition of substructure restoration occurring during swaging to 10 mm, the Cu lamellas of the 15 mm and 10 mm composites were subjected to more detailed investigations, which involved observations of FIB–prepared TEM lamellas from the 15 mm and 10 mm clad composites, as well as detailed SEM scanning and evaluation of CSL (coincidence site lattice) boundaries for the 10 mm composite.

**Figure 10:**
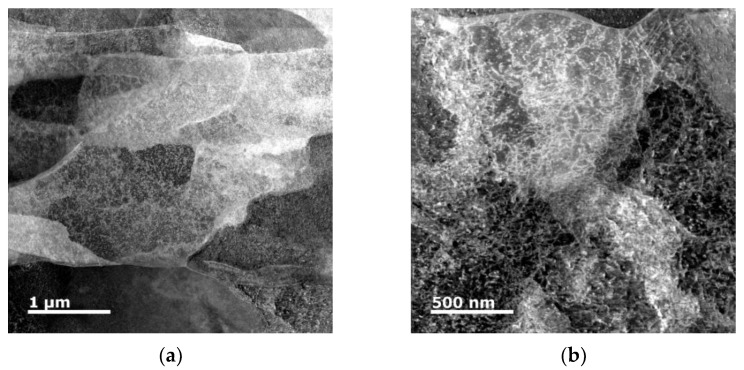

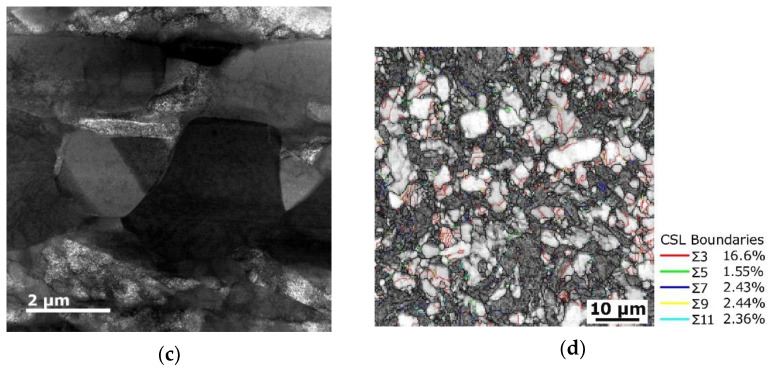
Scanning transmission electron microscopy (STEM) dark field (DF) images of Cu lamella of 15 mm composite–development of subgrains (**a**); accumulated dislocations (**b**). STEM DF image of Cu lamella of 10 mm composite–restored grains (**c**). Detailed SEM-EBSD scan of Cu lamella of 10 mm composite showing CSL boundaries (**d**).

Figure 10a,b show the scanning TEM (STEM) dark field (DF) images of substructure of a Cu lamella of the 15 mm composite. The figures clearly depict that the 15 mm composite featured a high density of accumulated dislocations as well as highly developed substructure. Figure 10c showing the STEM DF image of substructure of a Cu lamella of the 10 mm composite then shows that this conductor exhibited the presence of restored grains with a very low dislocations density. The 10 mm composite also featured the occurrence of deformation twins, which is documented by the CSL boundaries map, acquired via SEM-EBDS, depicted in Figure 10d. The image depicts the fractions of Σ3 (60°, <111>), Σ5 (36.87°, <100>), Σ7 (38.21°, <111>), Σ9 (38.94°, <110>), and Σ11 (50.48°, <110>) boundaries, which were observed within the structure of the Cu lamella.

### 3.3. Microhardness Measurements

**Figure 11:**
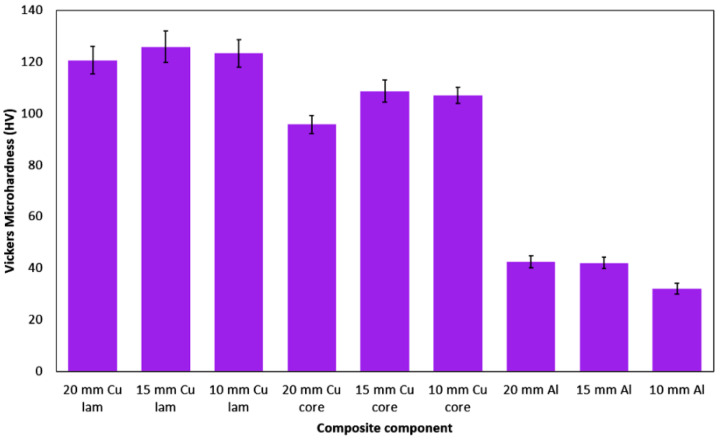
Vickers microhardness for individual components of swaged composites.

Last but not least, the microhardness of the individual components of the swaged clad composites was measured. The results of the measurements for the Cu lamellas, Cu cores, and Al matrices, together with depicted standard deviations, are summarized in Figure 11. The figure depicts that the highest HV values were detected for the Cu lamellas, while for the Cu cores they were slightly lower. As regards the composite diameters, the Cu components of the 15 mm composite featured the highest microhardness. Similar trend was also observed for the values of standard deviation (SD). The SD values were the highest for the Cu lamellas, for which they featured decreasing tendency with decreasing swaged rod diameter (from 6.07 for 20 mm composite to 5.21 for 10 mm composite). For the Cu cores, the SD values were slightly lower. However, for this composite diameter the highest SD value was detected for the 15 mm composite core (4.35). As regards the Al matrices, the HV values decreased with continuing swaging; for the 20 mm and 15 mm composites they were comparable (42.7 and 41 HV, respectively) and then decreased to 32.1 HV for the 10 mm composite. The Al matrices of all the examined composite diameters also exhibited the lowest SD values (2.42, 2.16, and 2.08 for the 20 mm, 15 mm, and 10 mm composite, respectively).

## 4. Discussion

The assembled Al–Cu semi-products with the original diameters of the outer Al matrix of 50 mm were room-temperature swaged down in multiple steps to the final clad conductors with the diameters of 20 mm, 15 mm, and 10 mm. Room-temperature processing prevented the development of intermetallic phases at the Al–Cu interfaces and ensured high-quality bonding of the individual composite components [13]. However, this non-negligibly affected the structures and electric properties of the composites, both of which are closely related to each other.

The analyses of the composites studied revealed that the swaging reductions down to 20 mm, 15 mm, and 10 mm imparted evident differences in the grain sizes between the axial Cu core, and peripheral Cu lamellas. In other words, the Cu lamellas of the clad composites featured smaller average grain sizes than their Cu cores despite the fact that the value of the average grain size for the individual Cu components decreased with swaging to smaller diameters. This phenomenon can be attributed to distribution of the imposed strain during rotary swaging. Through the effect of the swaging dies, the strain is imposed gradually from the periphery towards the axial region of the swaged rod. In addition, the imposed strain involves a portion of shear strain, the effect of which must be considered, too. In other words, swaging imparted intense grain refinement primarily in the (sub)peripheral regions of the composite. Continuous gradual swaging then enabled the effect of the imposed strain to penetrate deeper towards the axis of the swaged rod, which resulted in the introduction of (more) intense grain refinement also in the internal regions of the composite, and finally also in its axial region. The incremental character of the process thus influences the (sub) structure development not only for (commercially) pure electro-conductive metals, but also for composites. It was also the primary reason why the 10 mm composite exhibited dynamic recrystallization resulting in the presence of fine more or less equiaxed grains defined with HAGBs within the structure of the Cu components of this swaged composite. In other words, the energy accumulated during the gradual cold swaging reached its critical value during the swaging pass down to the diameter of 10 mm and invoked dynamic recrystallization.

Nevertheless, the (sub) structure development within the studied composites was also affected by several other factors, among which was different physical properties of the used metals, i.e., Al and Cu [42]. Given the different developments of deformation strengthening within Al and Cu, the increment of effective strain after a single swaging pass was different for both the component metals (manifested as differences in the average grain sizes of Cu components and Al matrices, and variations in values of microhardness). Another influencing factor was the plastic flow intensity. Despite the fact that the forward, i.e., axial, material flow is dominant during rotary swaging, its tangential and radial components have to also be considered [25]. For the composites, the plastic flow behaviour depends on the exact location, i.e., varies within the internal volumes of the component metals, and in the vicinities of Al–Cu interfaces. The differences in the plastic flow at mutual interfaces introduce additive shear strains, which can locally affect structures, but also the electric properties of the composites.

The results of the analyses of DC and AC characteristics (Table 2) showed that individual power losses (percentage of growth) between the individual swaging reductions were lower for the composite conductors than for Cu or Al conductors. In other words, the power losses observed after swaging from 20 mm to 15 mm (and from 15 mm to 10 mm) were lower for the clad conductor, than for the conductors from (commercially) pure metals. The most probable cause of this phenomenon was the observed (sub) structure development, i.e., the occurrence of structure obstacles affecting transfer of the electric current (presence of accumulated dislocations, deformation twinning, precipitation etc.). The structural observations also revealed that the conductors swaged to the diameters of 20 mm, 15 mm, and 10 mm exhibited differences in their DC and AC characteristics, regardless the conductor material (Al, Cu, or clad composite). This phenomenon can most probably be attributed to the studied *skin effect*. Therefore, the presence of structural obstacles has substantial effect on AC transfer characteristics and the occurrence of obstacles, such as grain boundaries, deformation twins, dislocations, or precipitates, in the (sub)peripheral regions of the conductor can emphasize differences between AC and DC characteristics of a composite conductor.

Rotary swaging, however, does not only impart strengthening via accumulation of structure defects and obstacles, but can also introduce restoration processes. During swaging to the diameters of 20 mm and 15 mm, the imposed strain was most probably primarily consumed by the Al matrix. This hypothesis is supported by the fact that the Cu components featured higher flow stress than the Al matrix (given by their different mechanical and physical properties) [42], but also by the presented findings. Among these were the increasing microhardness observed for the Cu components, but comparable microhardness observed for the Al matrices after swaging from 20 mm to 15 mm (indicating that the Al matrix consumed the majority of the imposed strain and exhibited restoration during the swaging pass), more or less unchanged texture intensity within the Cu lamellas but gradually decreasing texture intensity within the Al matrix, as well as relatively large average grain sizes and no presence of deformation twins in the Cu components of the 20 mm and 15 mm composites. In other words, the energy (effective imposed strain) imparted into the composite during swaging down to 15 mm was primarily transformed to axial plastic flow of the Al matrix and decrease in composite diameter.

On the other hand, final swaging to the diameter of 10 mm featured differences, especially as regards the behaviours of the Cu components of the clad composite. The results of the analyses showed that the structure exhibited the most evident grain refinement, together with the presence of deformation twins. For such structure changes to occur, the majority of the Al matrix needed to serve as a transmitter of imposed strain to the Cu composite components. This presupposition is supported by the observed decrease in the maximum texture intensity of the matrix, which was lower compared to the decrease in the maximum texture intensity occurring during swaging from 20 mm to 15 mm (see Figure 9d–f). Swaging to 10 mm also led to the development of restoration processes, which introduced restored equiaxed grains (Figure 7f), decrease in texture intensity and its randomization (Figure 9c), and decrease in dislocations density for the Cu components (Figure 10c). The restoration processes had eventually favourable effects on the AC characteristics of the conductor, as they compensated the increase in the volume of grain boundaries and presence of deformation twins. However, the difference between the AC and DC characteristics was negligible, since the *skin effect* is increasingly manifested by increasing the conductor diameters above 10 mm.

## 5. Conclusions

The study was focused on characterizing the (sub) structure development within innovative Al–Cu clad composite conductors with the diameters of 20 mm, 15 mm, and 10 mm prepared via room temperature rotary swaging, and correlating the occurring structural features with the behaviours of the conductors during transfer of alternating current (AC). The results of the analyses performed revealed that the proposed composite design affected favourably power losses occurring during AC transfer, as well as contributing to homogenization of current density across the cross-section of the swaged composite conductors, and thus contributed to a reduction in the undesirable influence of the *skin effect* on power losses (at least in comparison with a Cu conductor). Compared to direct current (DC) characteristics, the AC characteristics of the swaged composites featured increase in the resistance by 1.8%, 0.6%, and 0.1% for the 20 mm, 15 mm, and 10 mm composites. Incremental swaging to the diameters of 20 mm, 15 mm, and finally 10 mm introduced gradual grain refinement, texture randomization, and eventual structure restoration during the final swaging pass. Moreover, the rotary swaging imparted favourable increase in microhardness. The acquired results point to the conclusion that the presented design of the Al–Cu clad composite is favourable for AC transfer.

## Figures and Tables

**Table 1:** Characteristic values for used metals.

Material	Permeability *μ_r_* (-)	Specific Electric Resistivity *ρ* (Ω·m)·10^−9^	Skin Depth *δ* (m)·10^−3^
ETC Aluminium	1.000021	28.772	12.07
ETC Copper	0.999991	17.468	9.41

**Table 2:** Power losses during alternating current (AC) and direct current (DC) transfer.

Material	DC Power Losses *dP_DC_* (W)			AC Power Losses *dP_AC_* (W)			Coefficient *k_s_* (-)		
	D10	D15	D20	D10	D15	D20	D10	D15	D20
ETC Aluminium	3.664	1.631	0.919	3.666	1.636	0.928	1.001	1.003	1.009
ETC Copper	2.222	0.989	0.557	2.225	0.997	0.571	1.002	1.008	1.025
Clad composite	2.821	1.165	0.698	2.824	1.173	0.711	1.001	1.006	1.018

## Data Availability

The original data supporting the research is not publicly available but the data that is not confidential is available on request from the corresponding author.
